# CD55 deposited on synovial collagen fibers protects from immune complex-mediated arthritis

**DOI:** 10.1186/s13075-015-0518-4

**Published:** 2015-01-17

**Authors:** Olga N Karpus, Hans P Kiener, Birgit Niederreiter, A Seda Yilmaz-Elis, Jos van der Kaa, Valeria Ramaglia, Ramon Arens, Josef S Smolen, Marina Botto, Paul P Tak, J Sjef Verbeek, Jörg Hamann

**Affiliations:** Departments of Experimental Immunology, Internal Medicine, and Genetics, Room K0-140, Academic Medical Center, University of Amsterdam, Meibergdreef 9, 1105 AZ Amsterdam, The Netherlands; Department of Medicine III, Division of Rheumatology, Medical University of Vienna, Vienna General Hospital, Währinger Gürtel 18-20, A-1090 Vienna, Austria; Departments of Human Genetics and Immunohematology and Blood Transfusion, Leiden University Medical Center, Albinusdreef 2, 2333 ZA Leiden, The Netherlands; Centre for Complement & Inflammation Research, Department of Medicine, Imperial College London, South Kensington Campus, London, SW7 2AZ UK; GlaxoSmithKline Pharmaceuticals Research and Development, Gunnels Wood Road, Stevenage, SG1 2NY UK

## Abstract

**Introduction:**

CD55, a glycosylphosphatidylinositol-anchored, complement-regulating protein (decay-accelerating factor), is expressed by fibroblast-like synoviocytes (FLS) with high local abundance in the intimal lining layer. We here explored the basis and consequences of this uncommon presence.

**Methods:**

Synovial tissue, primary FLS cultures, and three-dimensional FLS micromasses were analyzed. CD55 expression was assessed by quantitative polymerase chain reaction (PCR), *in situ* hybridization, flow cytometry, and immunohistochemistry. Reticular fibers were visualized by Gomori staining and colocalization of CD55 with extracellular matrix (ECM) proteins by confocal microscopy. Membrane-bound CD55 was released from synovial tissue with phospholipase C. Functional consequences of CD55 expression were studied in the K/BxN serum transfer model of arthritis using mice that in addition to CD55 also lack FcγRIIB (CD32), increasing susceptibility for immune complex-mediated pathology.

**Results:**

Abundant CD55 expression seen in FLS of the intimal lining layer was associated with linearly oriented reticular fibers and was resistant to phospholipase C treatment. Expression of CD55 colocalized with collagen type I and III as well as with complement C3. A comparable distribution of CD55 was established in three-dimensional micromasses after ≥3 weeks of culture together with the ECM. CD55 deficiency did not enhance K/BxN serum-induced arthritis, but further exaggerated disease activity in *Fcgr2b*^−/−^ mice.

**Conclusions:**

CD55 is produced by FLS and deposited on the local collagen fiber meshwork, where it protects the synovial tissue against immune complex-mediated arthritis.

**Electronic supplementary material:**

The online version of this article (doi:10.1186/s13075-015-0518-4) contains supplementary material, which is available to authorized users.

## Introduction

Rheumatoid arthritis (RA) is a chronic, inflammatory disease of primarily the peripheral joints. The development of RA is characterized by transformation of the synovial tissue due to infiltration by reactive immune cells and profound changes in the resident synovial cells giving rise to inflammation, neovascularization, and hyperplasia, and leading finally to cartilage destruction, bone erosion, and functional impairment [1]. A unique cellular constituent of the synovial tissue is the fibroblast-like synoviocyte (FLS), a cell of mesenchymal origin that is closely connected to another synovial cell type of hematopoietic origin, known as the intimal macrophage [2,3]. FLS and intimal macrophages constitute the intimal lining layer of the joint. The healthy synovium comprises of two to three cell layers, which can increase to up to fifteen layers in RA. Subverting aberrant FLS proliferation, activation, and functional transformation [3] is at the basis of the intensive effort to treat the disease.

Recent studies using FLS placed into a preformed matrix and cultured as a floating sphere established that a large part of the behavior of these versatile cells is dependent on their three-dimensional organization, but independent of the presence of other mesenchymal or bone marrow-derived cell lineages [4,5]. Indeed, FLS micromasses develop a characteristic lining-sublining organization, synthesize lubricin, critical to the lubricating ability of the synovial fluid, and organize a basement membrane-like extracellular matrix (ECM), capable of supporting monocyte survival and compaction into the lining. When exposed to inflammatory stimuli, lining hyperplasia and remodeling occurs, thus, recapitulating the pathologic features of RA [5].

Our group and others previously described a highly abundant expression of CD55 by FLS in the intimal lining layer [6,7]. CD55 is a 70-kiloDalton (kDa) glycosylphosphatidylinositol (GPI)-anchored membrane protein possessing four short consensus repeat domains [8]. It is traditionally known to regulate activation of the immune system by facilitating the decay of the convertases that generate C3b and C5b (decay-accelerating factors), thereby protecting cells from the deleterious effects of complement activation [8]. In line with this well-established function, mice lacking CD55 are more susceptible to antibody-driven models of inflammatory diseases [9]. Another function of CD55 is its ability to bind the adhesion G protein-coupled receptor (GPCR) CD97 [10]. Adhesion GPCRs mediate cell-cell and cell-matrix interactions, and facilitate cell adhesion, orientation, migration and positioning in various developmental processes, in immunity, and in tumorigenesis [11]. We previously demonstrated that CD97, expressed by immune cells, binds CD55 in the intimal lining, implying a potential role in the infiltration of these cells [12]. Possibly related to the interaction of CD55 with CD97, we found amelioration of collagen-induced arthritis and a trend toward less severe K/BxN serum transfer arthritis in mice that lack CD55 or CD97 [13].

Its abundance and functional diversity has focused interest in the role of CD55 in synovial tissue. We recently tested the ability of proinflammatory cytokines and pathogen-associated molecular patterns to enhance CD55 expression in cultured FLS and found only a rather moderate upregulation by synthetic double-stranded ribonucleic acids (RNAs) [14]. Applying in-depth analysis of synovial tissue, three-dimensional FLS micromasses, and the K/BxN serum-induced experimental model of arthritis, we here explored the basis and the consequences of the enhanced presence of CD55 in the intimal lining layer. We demonstrate deposition of CD55 on a collagen fiber network that compacts the FLS and describe a supportive role of CD55 in protection from immune complex-mediated K/BxN serum transfer arthritis.

## Methods

### Collection and processing of synovial tissue

Synovial tissue samples were obtained by needle arthroscopy from patients with RA [15] who fulfilled the American College of Rheumatology/European League Against Rheumatism classification criteria [16]. The Medical Ethics Committees of the Academic Medical Center and Medical University of Vienna approved the study, and all patients gave written informed consent. Biopsy samples were snap-frozen in Tissue-Tek OCT (Miles Inc, Elkhart, IN, USA) immediately after collection. Cryostat sections (5 μm) were cut and stored at −80°C. Alternatively, synovial tissue obtained from synovectomy or joint replacement surgery was fixed in 4% paraformaldehyde in phosphate-buffered saline (PBS), dehydrated, paraffin-embedded, sectioned at 4-μm thickness, and stored at room temperature.

To remove GPI-linked proteins, thawed sections were washed two times in PBS and treated with 1 U/ml GPI-specific phospholipase C (Invitrogen, Bleiswijk, Netherlands) for 45 min at 37°C. Then, sections were fixed in acetone, washed with PBS, and stained as described below.

### Isolation, culture, and analysis of FLS

Single-cell suspensions were generated by finely mincing freshly isolated synovial tissue samples, followed by treatment with 0.5 mg/ml collagenase type VIII (Sigma-Aldrich, Zwijndrecht, Netherlands) for 2 h at 37°C. The obtained cell suspension was cultured in Dulbecco’s modified Eagle medium (DMEM, 1 g/l D-glucose; Invitrogen), supplemented with 10% fetal calf serum (FCS; GE Healthcare, Colbe, Germany), L-glutamine, HEPES, and antibiotics. Adhering cells were grown to subconfluence and split subsequently by trypsinization. Cells were used for experiments from passage 3 until passage 9 at 70 to 90% confluence. To generate hypoxic conditions, cells were cultured for 48 h at 1% O_2_. Control plates were incubated with 20% O_2_ (normoxia).

For flow cytometric analysis, cells were incubated for 30 min with anti-CD55-APC (clone IA10) or isotype control IgG2a-APC and analyzed on a FACSCalibur (all BD Biosciences, San Jose, CA, USA). Results were analyzed using FlowJo software (Tree Star Inc, Ashland, OR, USA).

For real-time polymerase chain reaction (PCR) analysis, total RNA was isolated using the Qiagen RNA extraction kit (Qiagen, Venlo, Netherlands). A total of 100 ng total RNA was reverse transcribed using oligo(dT), random hexamers, and M-MuLV reverse transcriptase from the First Strand cDNA Synthesis kit (Thermo Fisher Scientific, Waltham, MA, USA). Transcript levels of the hypoxia-regulated genes vascular endothelial growth factor (*VEGF*), leptin (*LEP*), and angiopoietin-like 4 (*ANGPTL4*) [17] were analyzed by quantitative PCR with the StepOnePlus Real-Time PCR system using Fast SYBR Green Master Mix (Applied Biosystems, Carlsbad, CA, USA). The following primer pairs (forward and reverse) were used: *VEGF* 5’-CTTGCCTTGCTGCTCTACCT-3′ and 5′-CTGCATGGTGATGTTGGACT-3′; *LEP* 5′-GGCTTTGGCCCTATCTTTTC-3′ and 5′-GGAATGAAGTCCAAACCGGTG-3′; *ANGPTL4* 5′-CCACTTGGGACCAGGATCAC-3′ and 5′-CGGAAGTACTGGCCGTTGAG-3′; 18S rRNA 5′-CGGCTACCACATCCAAGGAA-3′ and 5′-GCTGGAATTACCGCGGCT-3′. Gene transcription was normalized to 18S rRNA (ΔCt). The relative expression ratios were calculated using the 2^-ΔΔCt^ method.

### Preparation of three-dimensional micromass cultures

Micromass organ cultures were constructed as described elsewhere [4,5]. Briefly, FLS were released from the culture dish by trypsinization and resuspended in ice-cold Matrigel matrix (BD Biosciences) at a density of 3 to 5 × 10^6^ cells/ml. Droplets of the cell suspension (25 μl) were placed onto 12-well culture dishes, coated with poly-2-hydroxyethyl methacrylate (poly-HEMA; Sigma-Aldrich, Milwaukee, WI, USA) to prevent attachment of cells to the culture dish. Gelation was allowed for 30 min at 37°C. Thereafter, the FLS gel was overlaid with basal culture medium (DMEM with 10% FCS). The floating three-dimensional cultures were maintained for 6 weeks; the medium was routinely replaced twice weekly. For immunohistochemistry, all micromasses were fixed with 4% paraformaldehyde in PBS and embedded in paraffin.

For stimulation experiments, FLS-containing micromasses were cultured in basal medium or in basal medium containing 10 ng/ml tumor necrosis factor (TNF) (R&D Systems, Minneapolis, MN, USA) for the 14- to 28-day experimental course.

### Histology and immunohistochemistry

For (immuno)histochemical staining, paraffin sections were deparaffinized, rehydrated, and subjected to antigen retrieval by immersing them in sodium citrate buffer, pH 6.0, inside an 86°C-water bath. Endogenous peroxidase activity was blocked with 1% H_2_O_2_. Slides were incubated for 1 h with a primary anti-CD55 (clone 143–30; LifeSpan BioSciences, Seattle, WA, USA) or anti-collagen III antibody (clone III-53; Acris Antibodies, Herford, Germany), washed, incubated with a secondary biotinylated goat-anti-mouse antibody (Vector Laboratories, Burlingame, CA, USA), and developed using a horseradish peroxidase detection kit (VECTASTAIN Elite ABC Kit and DAB substrate; Vector Laboratories). Images were captured with an Axioscope 2 light microscope (Zeiss, Jena, Germany) and processed using CellF software (Olympus Soft Imaging Solutions, Münster, Germany). Gomori’s silver impregnation was performed as described previously [4,5].

For immunofluorescent staining, frozen slides were thawed, fixed in acetone, washed in PBS, and blocked with 10% normal human serum. Sections were incubated with primary fluorescein isothiocyanate (FITC)-labeled anti-CD55 antibody (clone IA10; BD Biosciences) overnight at 4°C, washed, and analyzed by confocal microscopy. To visualize colocalization of CD55 with other stromal/ECM markers, FITC-conjugated mouse anti-CD55 was combined with rat anti-ER-TR7 (Abcam, Uithoorn, Netherlands) or biotinylated rabbit anti-collagen I (Abcam) or biotinylated rabbit anti-collagen III (Abcam), or biotinylated mouse anti-CD90 (clone 5E10; Biolegend, Uithoorn, Netherlands). From every section, we took three to six confocal microscope images. Localization of C3b was detected using FITC-conjugated rabbit anti-human C3c (Dako, Heverlee, Belgium), which recognizes deposited C3b in tissue. After washing in PBS, tissue sections were stained with secondary fluorescent-labeled antibodies. All sections were mounted with Vectashield containing DAPI (Vector Laboratories). Photographs were taken with a TCS SP8 X confocal microscope, using X Pro imaging software (both from Leica Geosystems, Munich, Germany). Image processing was done using Photoshop software (Adobe Systems, San Jose, CA, USA).

### *In situ* hybridization

*In situ* hybridization for CD55 was performed in paraffin sections of synovial tissue using a 5′-fluorescein-labeled 19-mer locked nucleic acid (LNA) antisense oligonucleotide containing LNAs (capital letters) and 2′-O-methyl-RNAs (small letters): 5′-FAM-TauGccAccTggTacAucA-3′ (Ribotask ApS, Odense, Denmark). Sections were deparaffinized, boiled in 10 mM citrate buffer (pH 6) for 10 min, and hybridized at 60°C for 30 min in 50% (v/v) deionized formamide, 600 mM NaCl, 10 mM HEPES buffer (pH 7.5), 1 mM EDTA, 5 x Denhardt’s reagent, and 200 ug/ml denaturated herring sperm with 100 nM of CD55 oligonucleotide. After hybridization, tissue sections were washed for 5 min each in 2 x, 0.5 x, and 0.2 x standard saline citrate at 60°C. Hybridized oligonucleotides were detected by incubation for 1 h with 1:2000 dilution of AP-labeled anti-5’-fluorescein Fab fragments (Roche, Woerden, Netherlands) and visualization using Alkaline Phosphatase Substrate kit III (Vector Laboratories) and Nuclear Fast Red (Sigma-Aldrich) counterstaining.

### Mice and generation of bone marrow chimeras

*Fcgr2b*^−/−^ mice generated using C57BL/6 embryonic stem cells were previously described by us [18]. The generation of *Cd55*^−/−^ mice using C57BL/6 embryonic stem cells will be described elsewhere. *Cd55*^−/−^ mice were crossed with *Fcgr2b*^−/−^ mice in order to obtain *Fcgr2b*^−/−^ x *Cd55*^−/−^ mice. All animal experiments were approved by the Animal Experiment Committee of the Leiden University Medical Center.

Bone marrow chimeras were generated by lethal irradiation of recipient mice with 8 Gy for 10 min, followed by retro-orbital injection with 8 × 10^6^ nucleated donor bone marrow cells 6 h later. Injection of *Fcgr2b*^−/−^ mice with bone marrow cells from *Fcgr2b*^−/−^ mice resulted in *Fcgr2b*^−/−^ x *Cd55*^host+/+BM+/+^ mice, injection of *Fcgr2b*^−/−^ mice with bone marrow cells from *Fcgr2b*^*−/−*^ x *Cd55*^*−/−*^ mice resulted in *Fcgr2b*^−/−^ x *Cd55*^host+/+BM−/−^ mice, and injection of *Fcgr2b*^*−/−*^ x *Cd55*^*−/−*^ mice with bone marrow cells from *Fcgr2b*^*−/−*^ mice resulted in *Fcgr2b*^−/−^ x *Cd55*^host−/−BM+/+^ mice. During the 4 weeks after bone marrow transplantation, mice received drinking water containing antibiotics. To confirm bone marrow reconstitution, a few drops of blood were collected from the tail vein after 5 weeks and analyzed by flow cytometry for CD55 expression on T, B, and myeloid cells. All cells were confirmed to be of donor origin.

### K/BxN serum transfer model and evaluation of arthritis development

Serum was harvested from 5- to 6-week-old K/BxN arthritic mice and stored at −80°C until usage. Arthritis was induced in recipient animals by intraperitoneal injection (12.5 μl serum/g body weight) on day 0 and day 2. Disease development was evaluated over 10 days using an extended scoring protocol [19]. In short, arthritic toes and knuckles were scored as 1, arthritic ankles or mid paws were evaluated on a scale from 1 to 5; so, each limb obtained a score between 0 and 15. Each mouse therefore could reach a total score of 60. Ankle thickness of each hind paw was measured using a caliper (Mitutoyo, Aurora, IL, USA) and compared with ankle thickness of the same hind paw at day 0.

Mice were sacrificed on day 10. Right hind paws were dissected, fixed in 4% buffered formalin for 1 day, demineralized with Osteosoft (Merck, Darmstadt, Germany) for 21 days, and embedded in paraffin. Sagittal 5-μm tissue sections were stained with hematoxylin and eosin to evaluate level of inflammation and with toluidine blue to evaluate loss of proteoglycans from cartilage matrix. The severity of joint inflammation was determined in a blinded manner by two independent observers using an arbitrary score (0 to 3, where 0 = no cells; 1 = mild cellularity; 2 = moderate cellularity; 3 = maximal cellularity). The cartilage destruction was measured as proteoglycan loss (% destained of total cartilage) using Image J software [20]. Image processing was done using Photoshop.

### Preparation of single-cell suspensions and flow cytometry

A few drops of blood were collected from the tail vein into heparin. Erythrocytes were lysed with a buffer containing 155 mM NH_4_Cl, 10 mM KHCO_3_, and 1 mM EDTA, and the remaining cells were washed in PBA. Synovial tissue was isolated from the left knee by medial patellar ligament incision. Single-cell suspensions were obtained by digestion with Liberase TM (0.4 mg/ml; Roche) and DNAse I (1 mg/ml; Roche) for 15 min, followed by mashing the remaining tissue in RPMI/10% FCS through a 70-μm cell strainer. After enzymatic treatment, cells were washed with PBA and stained directly. Nonspecific binding of antibodies was blocked by adding 10% normal mouse serum and 2.5 μg/ml anti-CD16/32 (clone 2.4G2; BD Biosciences) for 30 min at 4°C. Cells were stained with fluorescent-labeled antibodies to CD3, CD4, CD8, Ly-6G, CD11b, CD19 (all eBiosciences), and CD55 (BD Biosciences) for 30 min at 4°C. Viable cells were gated by forward and side scatter pattern. Flow cytometric analysis was performed using a FACSCanto (BD Biosciences) and the FlowJo software package.

### Statistics

Differences between more than two groups of mice after arthritis induction were evaluated with repeated measures analysis of variance (ANOVA) with Bonferonni *post hoc* test. *P* values ≤0.05 were considered to be statistically significant.

## Results

### Abundant presence of CD55 is an *in situ* characteristic of the intimal lining layer of the synovium

**Figure 1:**
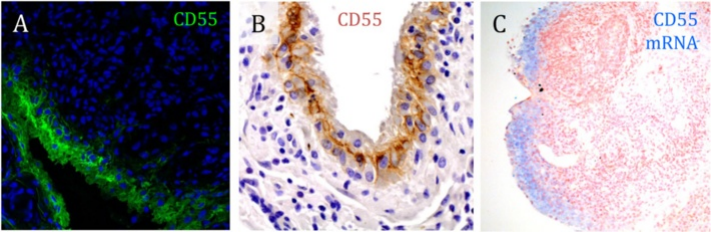
**CD55 is expressed in the intimal lining layer of rheumatoid arthritis (RA) synovial tissue. (A)** Sections of RA synovial tissue were stained with a fluorescein isothiocyanate (FITC)-labeled CD55 antibody (clone IA10) and analyzed by confocal microscopy. Note the fibrillar appearance of the fluorescent signal; magnification 20 x. **(B)** RA synovial tissue sections were stained with a CD55 antibody (clone 143–30) and visualized by immunohistochemistry and light microscopy; magnification 20 x. **(C)** *In situ* hybridization, using an antisense locked nucleic acid (LNA) oligomer, detects CD55 transcripts primarily in the synovial lining. Representative images are shown; magnification 10 x.

Immunofluorescence staining of RA synovial tissue revealed the characteristic, marked presence of CD55 in the intimal lining layer (Figure 1A). A strong staining was obtained with a FITC-labeled CD55 antibody without further signal amplification, closely matching results obtained with immunohistochemistry (Figure 1B). *In situ* hybridization with a CD55-specific antisense LNA oligomer generated a corresponding pattern, confirming that lining cells, previously identified as FLS [7], are the primary source of *CD55* gene expression in synovial tissue (Figure 1C). The hybridization signal of CD55 in the synovial sublining was weaker, yet detectable, which fits its wide cellular distribution, including most immune cells [21].

FLS, obtained from synovial tissue by enzymatic digestion, are widely used to study pathogenic mechanisms in RA. Notably, cultured FLS expressed rather moderate levels of CD55 mRNA, corresponding with quantities in peripheral blood mononuclear cells (Figure S1A in Additional file 1). We have recently tested the ability of proinflammatory cytokines and pathogen-associated molecular patterns to upregulate CD55 on cultured FLS and found that only double-stranded RNAs moderately enhanced its expression [14], not explaining the prominent presence in the intimal lining layer. CD55 is a hypoxia-induced gene in epithelial cells [22], and we reasoned that hypoxic conditions in the synovium [23] might contribute to its high intimal expression. However, culture of FLS at hypoxic conditions (1% O_2_) did not cause upregulation of CD55 mRNA or protein in FLS (Figure S1B/C in Additional file 1). We concluded that abundant expression of CD55 is a characteristic of the synovial intima that is not preserved in primary FLS cultures.

### Expression of CD55 in the intimal lining layer coincides with collagenous fibers

**Figure 2:**
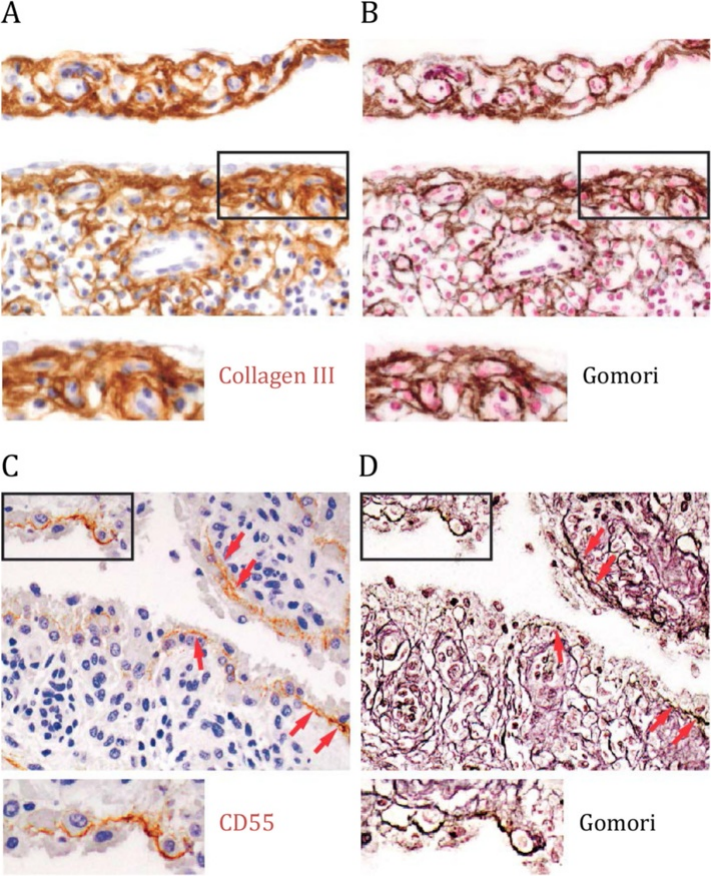
**Expression pattern of CD55 and collagen III coincides with collagenous structures in rheumatoid arthritis (RA) synovial tissue.** Sections of RA synovial tissue first were stained with **(A)** anti-collagen antibody or **(C)** anti-CD55 antibody and then processed to **(B/D)** Gomori silver impregnation. Red arrowheads indicate localization of CD55 to collagenous fibers. Shown are representative stainings derived by light microscopy; magnification 20 x.

Microscopy of synovial tissue revealed a distinct fibrillar staining pattern of CD55 (Figure 1A/B) indicating a possible extracellular distribution. To test this hypothesis, we stained paraffin sections of synovial tissue first with a CD55 or collagen III antibody and subsequently with Gomori’s silver technique, which visualizes reticular collagenous fibers [24,25]. The silver impregnation revealed a meshwork of fibers, a pattern closely matching the distribution of collagen III at both the intimal lining layer and the synovial sublining area (Figure 2A/B). At the intimal lining layer, CD55 staining coincided with these fibers (Figure 2C/D), suggesting localization of CD55 with collagenous fibers, specifically at the intima.

**Figure 3:**
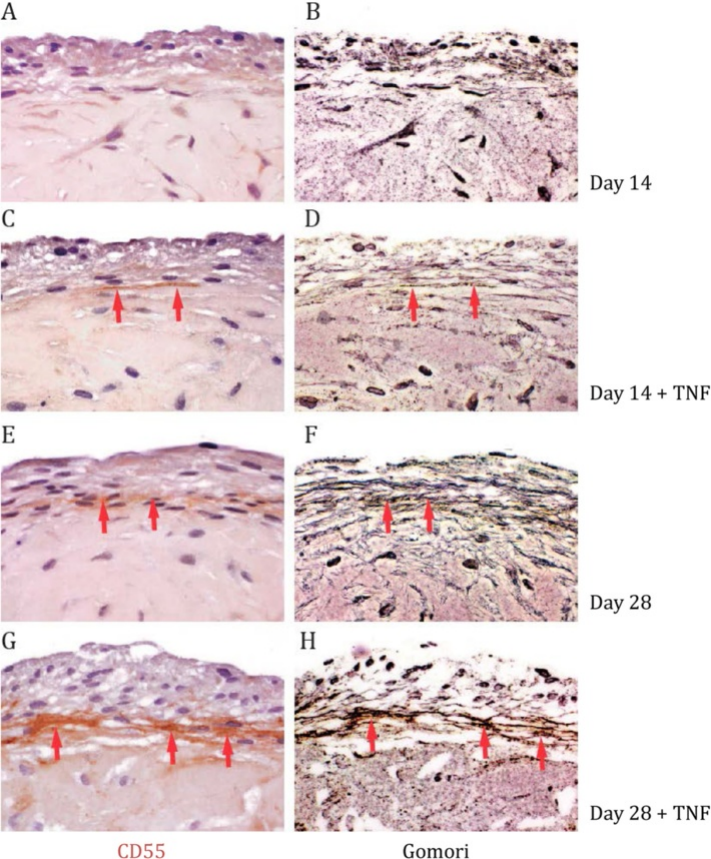
**Expression pattern of CD55 coincides with collagenous structures in three-dimensional fibroblast-like synoviocytes (FLS) micromasses. (A, B, E, F)** Sections of three-dimensional FLS micromasses were stained with anti-CD55 antibody at day 14 **(A)** and day 28 **(E)**, and then processed to Gomori silver impregnation (**B and F,** respectively). **(C, D, G, H)** Sections of three-dimensional FLS micromasses, which were cultured with 10 ng/ml tumor necrosis factor (TNF), were stained with anti-CD55 antibody at day 14 **(C)** and day 28 **(G)**, and then processed to Gomori silver impregnation (**D and H**, respectively). Red arrowheads indicate a similar distribution of CD55 and collagenous fibers. Shown are representative stainings derived by light microscopy; magnification 40 x.

Three-dimensional micromasses, grown from FLS, mimic phenotypic characteristics of the normal and the hyperplastic intimal lining layer [5]. When staining three-dimensional micromasses, we detected a CD55 signal after about 3 to 4 weeks of culture that was not visible at earlier time points (Figure 3A/E). As in synovial tissue, CD55 staining appeared as a fibrillar pattern within and at the basis of the intimal lining layer. Gomori silver staining of micromasses confirmed the development of reticular fibers within time (Figure 3B/F). Addition of TNF caused hyperplasia of the lining [5], coinciding with an intensified CD55 signal (Figure 3C/G) and more refined collagenous fibers visualized by Gomori silver staining and collagen III immunohistochemistry (Figure 3D/H and Figure S2 in Additional file 1). Thus, three-dimensional FLS micromasses develop over time the characteristic fibrillar CD55 expression pattern found in the intimal lining layer and, in parallel, a collagenous meshwork.

Cellular CD55 is attached to the plasma membrane by a GPI anchor, accessible to cleavage by phospholipase C [26]. ECM-attached CD55, in contrast, is not sensitive to phospholipase C cleavage [27,28]. Tissue sections, treated with phospholipase C prior to immunofluorescent staining, retained the signal for CD55, but not for CD90 (Thy-1), which also is a GPI-anchored molecule (Figure S3 in Additional file 1). Therefore, we concluded that the majority of CD55 in the synovial tissue is deposited extracellularly.

### Molecular composition of CD55^+^ collagenous fibers

**Figure 4:**
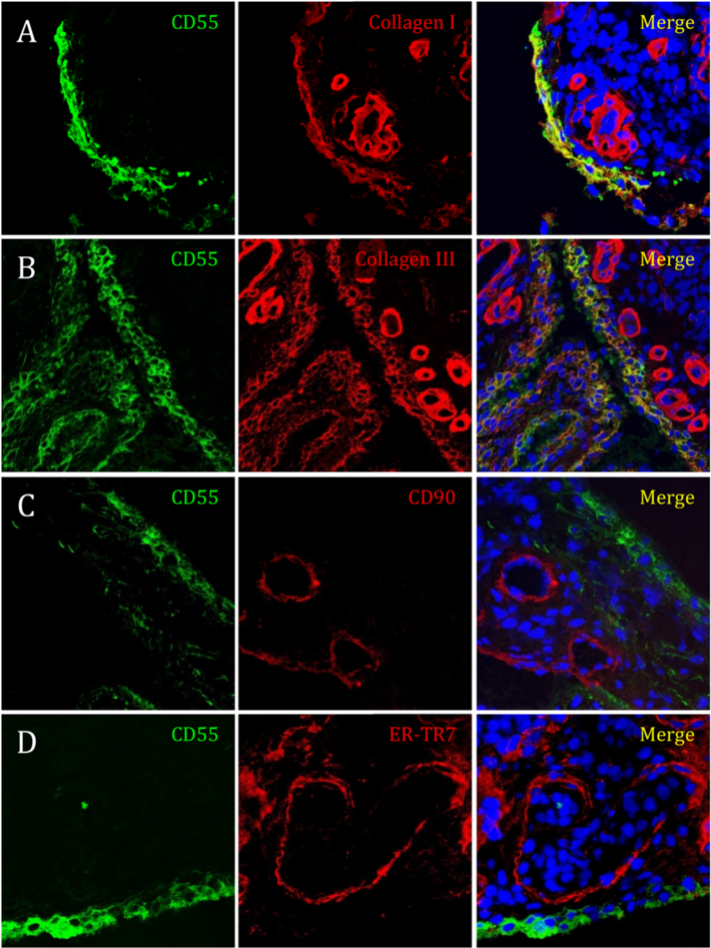
**CD55 colocalizes with collagen I and collagen III, but not with ER-TR7 or CD90 in rheumatoid arthritis (RA) synovial tissue.** Sections of RA synovial tissue were costained for CD55 (green) and **(A)** collagen I, **(B)** collagen III **(C)** CD90, or **(D)** ER-TR7 (all red, overlay yellow) and analyzed by confocal microscopy. Shown are representative stainings, magnification 63 x.

Silver impregnation techniques, like Gomori’s method, are widely used to detect reticular fibers. However, they have no biochemical definition and cannot be used as staining techniques for reticulin or different types of collagen [29]. We therefore performed immunofluorescent double staining of CD55 together with collagen I and III, CD90, and ER-TR7 on RA synovial tissue sections. We detected a clear colocalization of CD55 with collagen I and collagen III in the intimal lining layer, but not the vasculature, localized in the synovial sublining (Figure 4A/B and Figure S4A/C in Additional file 1). The latter was confirmed by costaining CD55 with CD90, a marker expressed by endothelial cells (Figure 4C).

Collagens are constituents of collagen fibers; however, collagen III also builds the core of reticular fibers [24,25]. ER-TR7 is an antigen of unknown origin expressed by fibroblast-like reticular cells [30] and used commonly to visualize the reticular network in lymph nodes [31,32]. We found an ER-TR7^+^ network in the synovial sublining that did not overlap with the CD55^+^ collagenous fibers in the lining (Figure 4D and Figure S4B in Additional file 1). Thus, deposition of CD55 is a distinct characteristic of collagenous fibers in the intimal lining layer, but not of the ER-TR7^+^ network in the sublining.

The unique localization of CD55 at the edge of the synovial tissue, conjugated to collagenous fibers, implied a possible role of CD55 in protecting the synovial tissue from attack by complement. Confirming previous reports [33,34], we detected C3b in the intimal lining layer with a distribution similar to CD55 (Figure S5 in Additional file 1).

### CD55 cooperates with FcγRIIB (CD32) in protecting against immune complex-mediated arthritis

**Figure 5:**
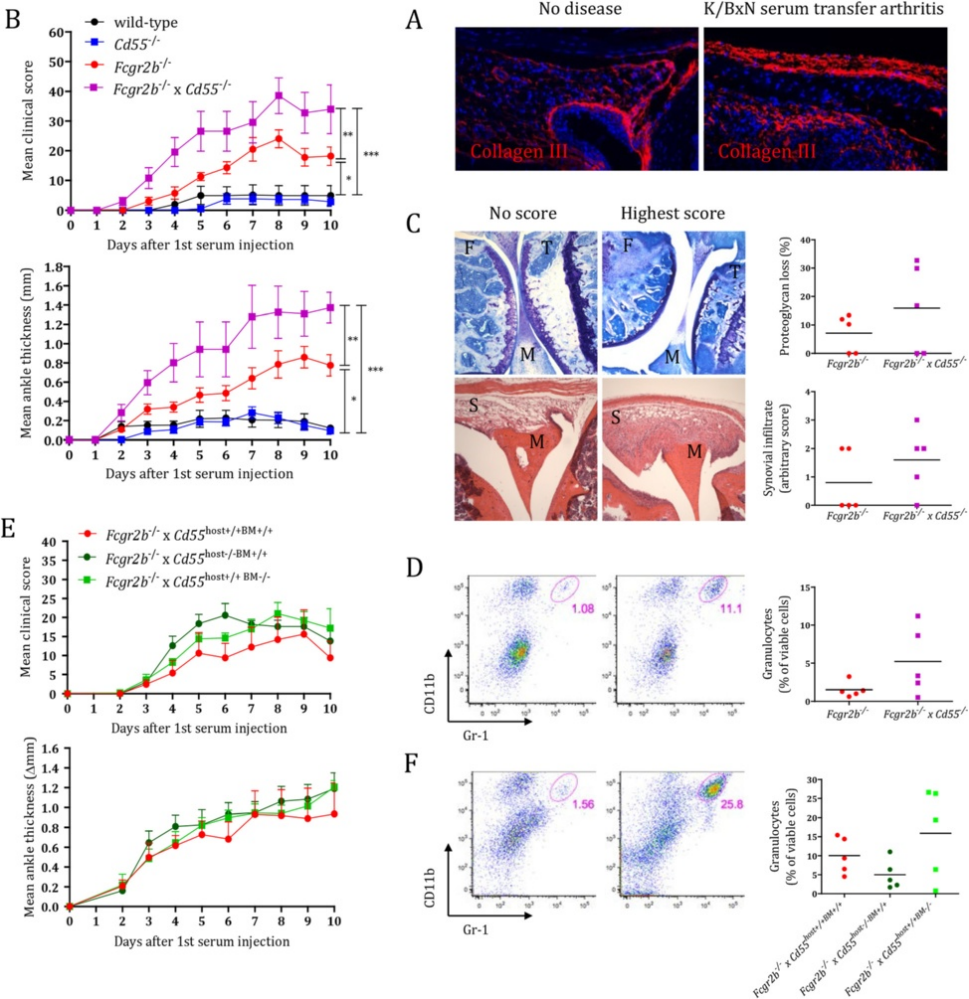
**CD55 either on immune or stromal cells protects from K/BxN serum transfer-induced arthritis in the absence of FcγRIIB (CD32).** Mice were injected at day 0 with 12.5 μl/g K/BxN serum and followed for disease development. At day 10, animals were sacrificed for further analysis. **(A)** Collagen III staining in representative knee joint sections of non-diseased and arthritic *Fcgr2b* ^−/−^ mice. Magnification 20 x. **(B)** Development of arthritis evaluated by measuring clinical scores and ankle thickness. Depicted are mean ± standard error of the mean (SEM) of five animals per group. ^***^, *P* <0.001; ^**^, *P* <0.01; ^*^, *P* <0.05 **(C)** Histological evaluation of knee joints for proteoglycan loss (toluidine blue; above) and cell infiltration (hematoxylin and eosin; below). Photographs are representative of mice with no or highest disease activity in the experiment, respectively. Joint structures are labeled as F, femur; M, meniscus; S, synovium; T, tibia. Magnification 5 x. **(D)** Flow cytometry plots of isolated synovial tissue cells stained for CD11b^+^Gr1^+^ granulocytes, representative of mice with no or highest disease activity in the experiment, respectively. **(E)** Development of arthritis evaluated by measuring clinical scores and ankle thickness in bone marrow chimeric mice that lacked CD55 on stromal cells (*Fcgr2b* ^−/−^ x *Cd55* ^host−/−BM+/+^, depicted as a dark green line), on immune cells (*Fcgr2b* ^−/−^ x *Cd55* ^host+/+BM−/−^, depicted as a light green line), or on none of the two compartments (*Fcgr2b* ^−/−^ x *Cd55* ^host+/+BM+/+^, depicted as a red line). Depicted are mean ± SEM values of five animals per group. **(F)** Flow cytometry plots of isolated synovial tissue cells stained for CD11b^+^Gr1^+^ granulocytes, representative of mice with no or highest disease activity in the experiment, respectively. Quantifications provided to the right in **(C)**, **(D)**, and **(F)** show individual mice (dots) with a horizontal line indicating the mean per group (n = 5).

To address the possibility that CD55 protects the synovial tissue from complement-mediated immune activation *in vivo*, we studied the K/BxN serum transfer model of arthritis. The K/BxN model is driven by immune complexes and depends on complement, Fc receptors, and innate immune cells, mainly neutrophils [35-37]. In analogy with the situation seen in RA synovial tissue, we observed intensive collagen III staining in the intimal lining layer in arthritic mice (Figure 5A).

Notably, we previously reported a slight protection of mice lacking CD55 in the K/BxN model, which mirrored a similar phenotype in mice lacking the CD55 interacting partner CD97 [13]. In the current study, we used less potent serum to have a higher threshold for disease development. In this setting, mice lacking CD55, like wild-type mice, did not develop arthritis (Figure 5B). In contrast, mice lacking FcγRIIB (*Fcgr2b*^−/−^) were susceptible, which fits with the crucial role of this inhibitory receptor in the control of immune complex-mediated inflammation [18]. When we crossed *Cd55*^−/−^ mice with *Fcgr2b*^−/−^ mice, compound animals developed significantly more severe disease than mice that only lack FcyRIIB (Figure 5B). Corresponding herewith, *Fcgr2b*^−/−^ x *Cd55*^−/−^ mice showed a trend toward enhanced cartilage destruction and immune cell infiltration (Figure 5C) with more Gr1^+^CD11b^+^ granulocytes present locally in the synovial tissue (Figure 5D). Thus, combined absence of FcγRIIB receptor and CD55 deteriorates K/BxN serum-induced arthritis.

CD55 is widely expressed by endothelial, immune, and stromal cells [8,18]. To explore the role of stromal CD55 in arthritis development, as an extrapolation to human CD55, abundantly expressed by FLS, we generated bone marrow chimeric mice expressing CD55 on either immune or non-immune cells. We found that *Fcgr2b*^−/−^ x *Cd55*^host+/+BM−/−^ and *Fcgr2b*^−/−^ x *Cd55*^host−/−BM+/+^ mice were equally susceptible to K/BxN serum transfer arthritis compared to *Fcgr2b*^−/−^ x *Cd55*^host+/+BM+/+^ mice (Figure 5E) with a tendency toward slightly higher disease scores and granulocyte infiltration in synovial tissue (Figure 5E/F). We concluded that CD55 on both immune and stromal cells is contributing to protection against immune complex-mediated arthritis in susceptible mice.

## Discussion

We here report that CD55, produced by FLS, is deposited in large amounts at a collagenous fiber network within and at the basis of the intimal lining layer. Fiber networks building the ECM provide structural support for organs and tissues, for cell layers in the form of basement membranes and for individual cells as substrates for migration. Moreover, they are essential for the binding, transport, and presentation of growth factors, inflammatory signals, chemoattractants, and soluble antigens that govern the differentiation, proliferation, survival, polarity, and migration of immune cells [38]. The synovial tissue comprises various ECM structures [39], which are synthesized by FLS. More recent studies with three-dimensional micromasses unveiled that FLS are capable of autonomously establishing a complex basement membrane-like structure that enables the intimal lining layer-synovial sublining architecture as well as fibrilliar meshworks in lining and sublining [5].

Deposition of CD55 on structures of the ECM was first reported by Medof and coworkers, who noted a prominent fibrillar staining in fibrous sheaths surrounding myocardial muscle bundles, in interstitium underlying the endocardium, and in connective tissue adjacent to the synovium [40]. Later studies confirmed and extended these findings by describing CD55 attached to subendothelial collagenous fibers underneath the vasculature [27] and various epithelia [41], as well as to elastic fibers surrounding nerve plexuses of the enteric nervous system [28]. Notable is the expression of CD55 in the stroma of various tumors. Niehans and colleagues described deposition of large amounts of CD55 in stroma surrounding infiltrating (adeno)carcinoma of the breast, colon, kidney, and lung, while normal tissue stroma showed only small, localized deposits [41]. In line herewith, CD55 overexpression has been a target for intestinal tumor imaging, conferring with a poor prognosis in colorectal cancer patients [42]. Together, these findings suggest that abnormal expression of CD55 in the human body, as seen in tumors and the arthritic intimal lining layer, coincides with its deposition on ECM structures.

The collagenous ECM system comprises different meshworks [24,25]. By studying RA synovial tissue and three-dimensional RA FLS micromasses, we here show that CD55 in synovial tissue is deposited on collagen type I^+^/III^+^ fibers, which are restricted to and established along with the intimal lining, where FLS are compacted. Despite visualization of these fibers by silver impregnation, they did not stain for ER-TR7, a marker widely used to identify reticular networks [24,25]. Notably, ER-TR7 antibody generated a widespread staining pattern, almost inverse to the CD55^+^ network, in the synovial sublining. Whether this ER-TR7 network, in line with its role in secondary lymphoid organs [31,32], facilitates ectopic lymphoid neogenesis in synovial tissue remains to be investigated. Our data imply the existence of different fiber meshworks in the synovial tissue, which may serve distinct functions.

The mechanism of CD55 deposition on synovial ECM remains to be established. Of note, development of the CD55^+^ collagenous network was enhanced in micromasses grown with TNF, demonstrating that FLS respond to proinflammatory stimuli by expanding the intimal lining layer including its ECM components. This finding matches observations that formation of the reticular network by fibroblast reticular cells in lymph nodes requires TNF receptor engagement [31], suggesting an essential role of TNF in the development of different collagenous meshworks. Anti-TNF therapy may affect this system, providing an additional mechanism of its action [43].

Previous studies by Hindmarsh and Marks showed that complement activation occurs on ECM and can be controlled by matrix-bound CD55 [27,44]. The need for an effective control of complement in the synovium is evident from studies showing consumption of complement components and generation of complement activation products in the synovial fluid of RA patients [45-47]. Moreover, C3 is deposited on the surface of cartilage and synovium in RA [33,34] and in experimental models of arthritis [48,49]. Circulating C3 is necessary and sufficient for antibody-driven K/BxN serum transfer arthritis [36]. Nevertheless, in different settings of this model, studied here and previously by our group [13], deletion of CD55 did not aggravate arthritis activity. In contrast, lack of CD55 further enhanced disease activity in *Fcgr2b*^−/−^ mice, which are highly susceptible to the K/BxN serum transfer model [18]. The adverse effect of CD55 deficiency in compound animals was not seen in bone marrow chimeric mice that expressed CD55 either on immune or non-immune cells, indicating that CD55 on both cellular compartments contributes to the control of complement activation.

Immune complex-mediated inflammation is mediated by a molecular partnership between Fc-gamma receptors (FcγRs), complement receptors, and additional modulators, such as C-type lectins [50]. Complement activation generates C5a, which by binding to its receptor (C5aR; CD88) lowers the threshold for FcγR activation through changing the ratio of expression of activating (FcγRI and FcγRIII) and inhibitory (FcγRIIB) receptors [51]. Conversely, immune complexes binding to FcγR enhance the synthesis of C5 and, consequently, promote the generation of C5a. This positive-feedback loop [52] essentially contributes to the pathogenesis of inflammatory disorders; however, the factors that tip the balance toward disease have remained elusive, and little is known regarding the mechanisms that circumvent C5a-induced excessive tissue immune activation and damage [52]. Our study suggests that CD55, which accelerates the decay of C3 convertases [8], provides a safety mechanism that is of critical importance once the activating-inhibitory FcγR ratio turns toward more activation.

## Conclusions

Our findings suggest that the abundant presence of CD55 in the intimal lining layer of the synovial tissue is due to deposition on a local meshwork of collagenous fibers that compacts the cellular compartment. Moreover, this study helps clarify the roles of CD55 in the pathogenesis of arthritis by demonstrating involvement in the protection against immune complex-mediated K/BxN serum transfer arthritis in susceptible mice lacking the inhibitory FcγRIIB.

## Additional file

Additional file 1: Figure S1. Cultured FLS express average levels of CD55. **Figure S2.** Expression pattern of collagen III in three-dimensional FLS micromasses coincides with collagenous structures visualized by Gomori silver staining. **Figure S3.** CD55, deposited in the extracellular matrix of the synovial lining, is resistant to phospholipase C treatment. **Figure S4.** Collagen I in synovial tissue colocalizes with CD55, ER-TR7, von Willebrand factor. **Figure S5.** Collagen I in synovial tissue colocalizes with complement component C3b.

## Abbreviations

DMEM: Dulbecco’s modified Eagle medium
ECM: extracellular matrix
FCS: fetal calf serum
FcγRs: Fc-gamma receptors
FITC: fluorescein isothiocyanate
FLS: fibroblast-like synoviocytes
GPCR: G protein-coupled receptor
GPI: glycosylphosphatidylinositol
kDa: kiloDalton
LNA: locked nucleic acid
PBS: phosphate-buffered saline
PCR: polymerase chain reaction
RA: rheumatoid arthritis
RNA: ribonucleic acid
TNF: tumor necrosis factor
