# Supplementary material for: CD55 deposited on synovial collagen fibers protects from immune complex-mediated arthritis
Source: Arthritis Res Ther. 2015 Jan 17;17(1):6. doi: 10.1186/s13075-015-0518-4 (PMC4325944; doi:10.1186/s13075-015-0518-4)
Supplement: Additional file 1: Figure S1. — Cultured FLS express average levels of CD55. Figure S2. Expression pattern of collagen III in three-dimensional FLS micromasses coincides with collagenous structures visualized by Gomori silver staining. Figure S3. CD55, deposited in the extracellular matrix of the synovial lining, is resistant to phospholipase C treatment. Figure S4. Collagen I in synovial tissue colocalizes with CD55, ER-TR7, von Willebrand factor. Figure S5. Collagen I in synovial tissue colocalizes with complement component C3b. [file 13075_2015_518_MOESM1_ESM.pdf]

Supplemental Information

CD55 deposited on synovial collagen fibers protects from immune complex-mediated arthritis

Olga N. Karpus, Hans P. Kiener, Birgit Niederreiter, A. Seda Yilmaz-Elis, Valeria Ramaglia, Jos van der Kaa, Ramon Arens, Josef S. Smolen, Marina Botto, Paul P. Tak, J. Sjef Verbeek, and Jörg Hamann

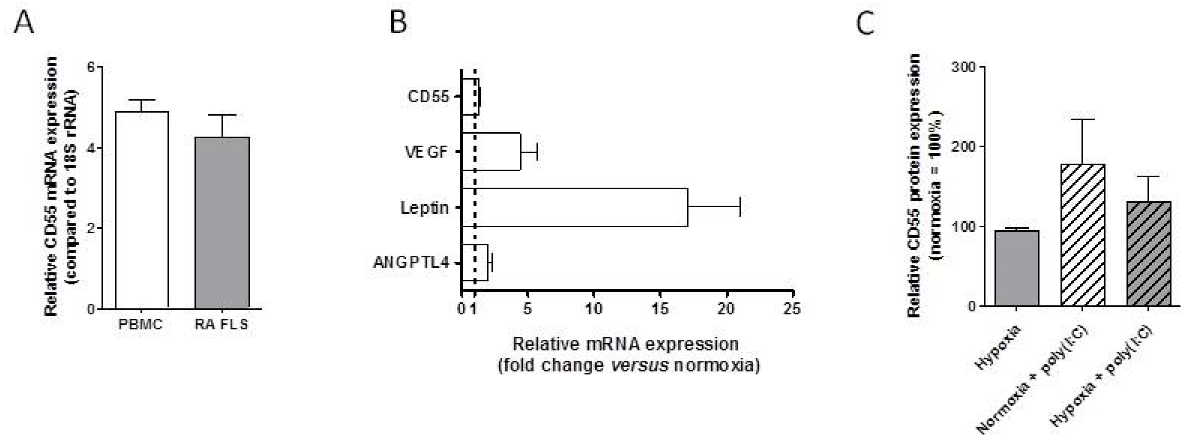

Figure S1. Cultured FLS express average levels of CD55. (A) Cultured RA FLS (n=6) express CD55 mRNA levels comparable to immune cells (PBMC) from healthy donors (n=3) as revealed by qPCR. (B) Hypoxic conditions do not enhance CD55 mRNA expression, quantified by qPCR, but increases transcription of the hypoxia-regulated genes VEGF (vascular endothelial growth factor), LEP (leptin), and ANGPTL4 (angiopoietin-like 4) (n=3). (C) dsRNA but not hypoxic conditions moderately induce surface expression of CD55 on FLS measured by flow cytometry (n=3).

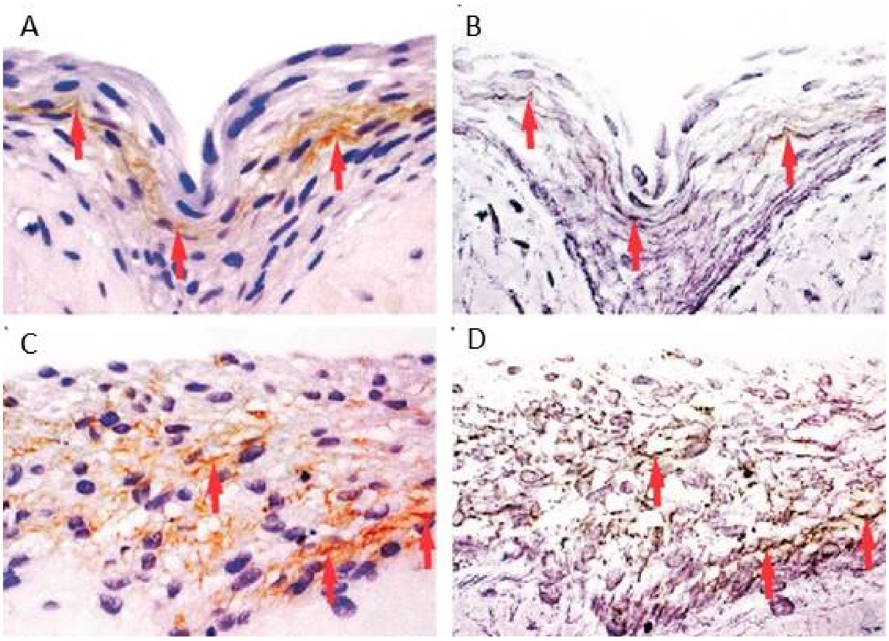

Figure S2. Expression pattern of collagen III in 3D FLS micromasses coincides with collagenous structures visualized by Gomori silver staining. (A-B) Sections of 3D FLS micromasses that were cultured for 28 days without (A-B) or with (C-D) 10 ng/ml TNF were stained with anti-collagen III antibody (A, C) and then processed to Gomori silver impregnation (B and D, respectively). Red arrowheads indicate a similar distribution of collagen III and collagenous fibers. Shown are representative stainings derived by light microscopy; magnification 40 x.

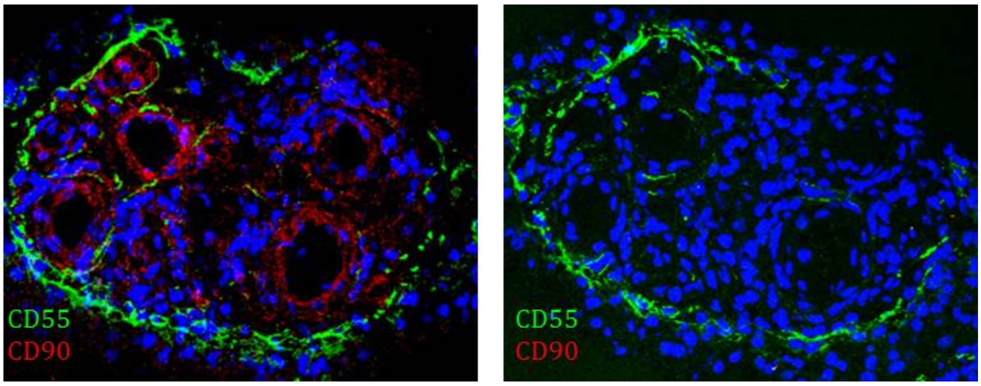

Figure S3. CD55, deposited in extracellular matrix of the synovial lining, is resistant to phospholipase C treatment. Tissue sections were left untreated (left panel) or incubated with phospholipase C (right panel) and subsequently stained for CD55 (green signal) and CD90 (red signal). In contrast to CD90, the CD55 signal is remained intact after enzymatic treatment. Shown are representative photographs derived by confocal microscope; magnification 63 x.

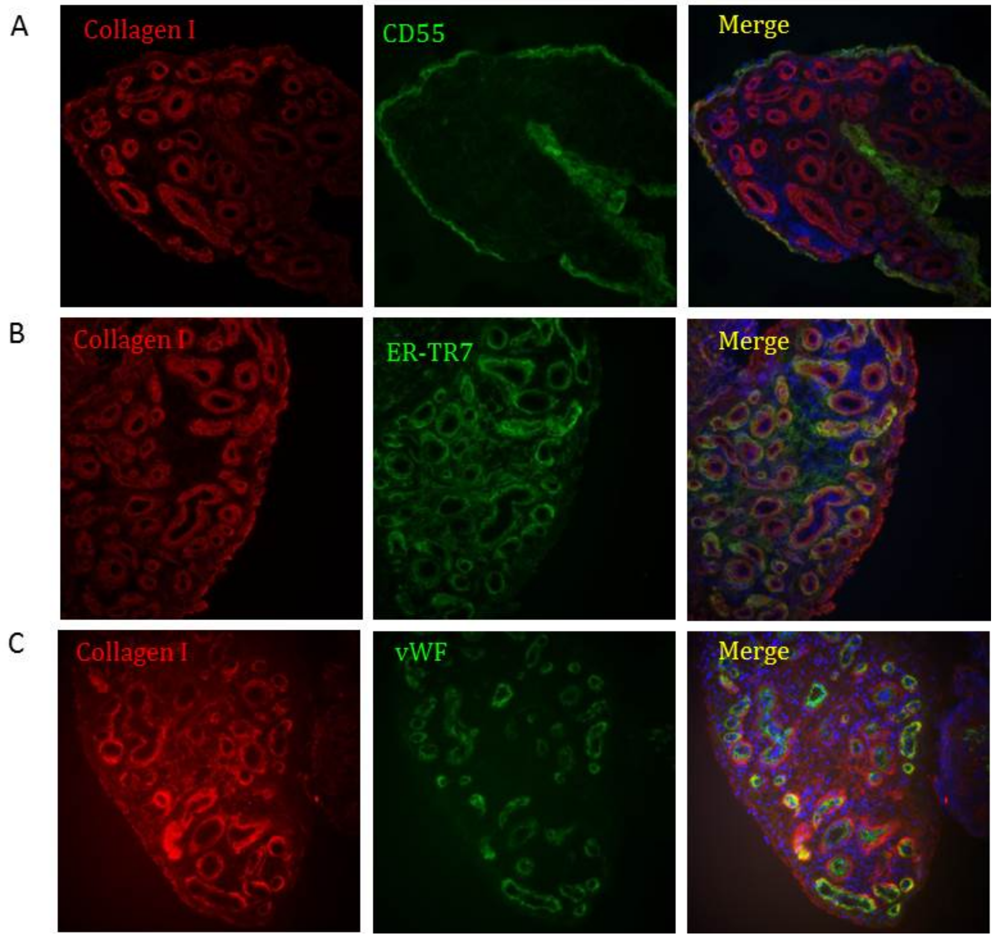

Figure S4. Collagen I in synovial tissue colocalizes with CD55, ER-TR7, von Willebrand factor. Sections of RA synovial tissue were costained for collagen I (red) and (A) CD55, (B) ER-TR7, or (C) von Willebrand factor (vWF) (all green, overlay yellow) and analyzed by fluorescent microscopy. Shown are representative photographs; magnification 20 x.

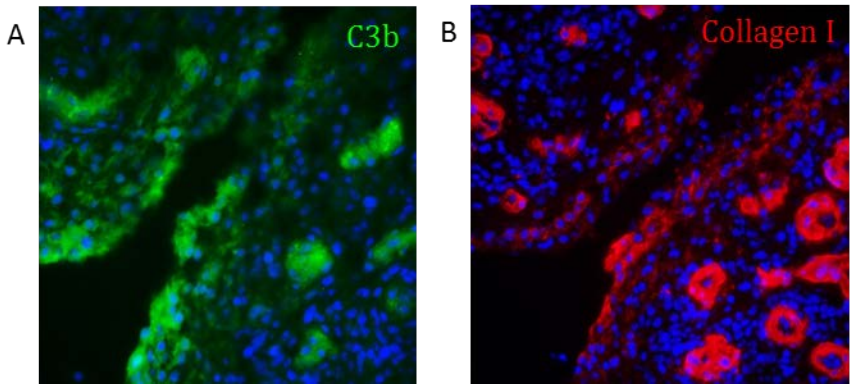

Figure S5. Collagen I in synovial tissue colocalizes with complement component C3b. Sequential sections of RA synovial tissue were stained with for C3b (A) or collagen I (B) and analyzed by fluorescent microscopy. Shown are representative photographs; magnification 40 x.
